# Revising the structure of a new eicosanoid from human platelets to 8,9–11,12-diepoxy-13-hydroxyeicosadienoic acid

**DOI:** 10.1074/jbc.RA119.008915

**Published:** 2019-05-06

**Authors:** Andrei Kornilov, Paul D. Kennedy, Maceler Aldrovandi, Andrew J. A. Watson, Christine Hinz, Bryan Harless, Joseph Colombo, Kirk M. Maxey, Victoria J. Tyrrell, Matthew Simon, Varinder K. Aggarwal, William E. Boeglin, Alan R. Brash, Robert C. Murphy, Valerie B. O'Donnell

**Affiliations:** From ‡ Cayman Chemical, Ann Arbor, Michigan 48108,; § Systems Immunity Research Institute, School of Medicine, Cardiff University, Cardiff CF14 4XN, United Kingdom,; ¶ School of Chemistry, University of Bristol, Bristol BS6 6RU, United Kingdom,; ‖ Department of Pharmacology and the Vanderbilt Institute of Chemical Biology, Vanderbilt University School of Medicine, Nashville, Tennessee 37232, and; ** Department of Pharmacology, University of Colorado, Aurora, Colorado 80045

**Keywords:** eicosanoid, cyclooxygenase (COX), platelet, lipid, lipid metabolism, 8,9–11,12-diepoxy-13-hydroxyeicosadienoic acid (8,9–11,12-DiEp-13-HEDE), 8-hydroxy-9,11-dioxolane eicosatetraenoic acid (DXA3), DiEpHEDE, immunity, leukocyte-regulating lipid

## Abstract

Eicosanoids are critical mediators of fever, pain, and inflammation generated by immune and tissue cells. We recently described a new bioactive eicosanoid generated by cyclooxygenase-1 (COX-1) turnover during platelet activation that can stimulate human neutrophil integrin expression. On the basis of mass spectrometry (MS/MS and MS^3^), stable isotope labeling, and GC-MS analysis, we previously proposed a structure of 8-hydroxy-9,11-dioxolane eicosatetraenoic acid (DXA_3_). Here, we achieved enzymatic synthesis and ^1^H NMR characterization of this compound with results in conflict with the previously proposed structural assignment. Accordingly, by using LC-MS, we screened autoxidation reactions of 11-hydroperoxy-eicosatetraenoic acid (11-HpETE) and thereby identified a candidate sharing the precise reverse-phase chromatographic and MS characteristics of the platelet product. We optimized these methods to increase yield, allowing full structural analysis by ^1^H NMR. The revised assignment is presented here as 8,9–11,12-diepoxy-13-hydroxyeicosadienoic acid, abbreviated to 8,9–11,12-DiEp-13-HEDE or DiEpHEDE, substituted for the previous name DXA_3_. We found that in platelets, the lipid likely forms via dioxolane ring opening with rearrangement to the diepoxy moieties followed by oxygen insertion at C13. We present its enzymatic biosynthetic pathway and MS/MS fragmentation pattern and, using the synthetic compound, demonstrate that it has bioactivity. For the platelet lipid, we estimate 16 isomers based on our current knowledge (and four isomers for the synthetic lipid). Determining the exact isomeric structure of the platelet lipid remains to be undertaken.

## Introduction

Platelets regulate innate immunity during acute infection and injury through their interactions with leukocytes (1–3). These cells generate lipid mediators, including eicosanoids such as thromboxane A_2_, 12-hydroxyeicosatetraenoic acid, and lesser amounts of prostaglandins (PGs)^3^ such as PGE_2_.

Recently, we examined whether platelets release leukocyte-regulating lipids using a lipidomic approach and uncovered a new eicosanoid formed via COX-1 or COX-2 oxidation of arachidonate (4, 5). Based on extensive biochemical evidence, including MS/MS of the platelet lipid compared with synthetically generated stable isotope-labeled analogs, the structure of 8-hydroxy-9,11-dioxolane eicosatetraenoic acid (DXA_3_) was initially proposed. We also used GC-MS and demonstrated generation of an analogous lipid using purified recombinant COX isoforms. A pathway to chemically synthesized lipid via 11-HpETE oxidation was shown, consistent with previous reports for cholesteryl dioxolanes (6, 7). A structure and mechanism of formation were suggested based on our findings. At that time, we did not have sufficient quantities of purified synthetic lipid for NMR and had to rely on analysis of semipurified synthetic lipid from low-yield reactions only.

To obtain more definitive evidence, we next attempted to generate the proposed dioxolane structure using total synthesis approaches. However, this was not successful. Instead, herein, this was achieved through enzymatic oxidation of preformed EETs to form two candidate molecules. However, NMR analysis of these found that dioxolane lipids did not match the platelet isomer. As an alternative strategy, we next focused on modifying and scaling up the chemical oxidation approach to yield a lipid that is consistent with the platelet isomer in terms of MS/MS and reverse-phase retention time. We refer to this herein as the “synthetic” lipid (which contains one primary lipid and small amounts of two additional isomers). Once isolated, ^1^NMR and COSY analyses were undertaken. The nature of functional groups was revealed, along with position and geometry for both epoxides and one double bond. These data are presented in full herein. In summary, based on our data, the “platelet” lipid is proposed as a further stable metabolite of COX-1, generated likely via ring opening of the dioxolane ring followed by rearrangement and further oxygen insertion. The new structure is presented here as 8,9–11,12-DiEp-13-HEDE (DiEpHEDE), a diepoxy-monohydroxyeicosadienoic acid, which is generated by COX-1 in human platelets.

## Results

### Synthesis and purification of DXA_3_ from oxidation of EETs

**Figure 1.**
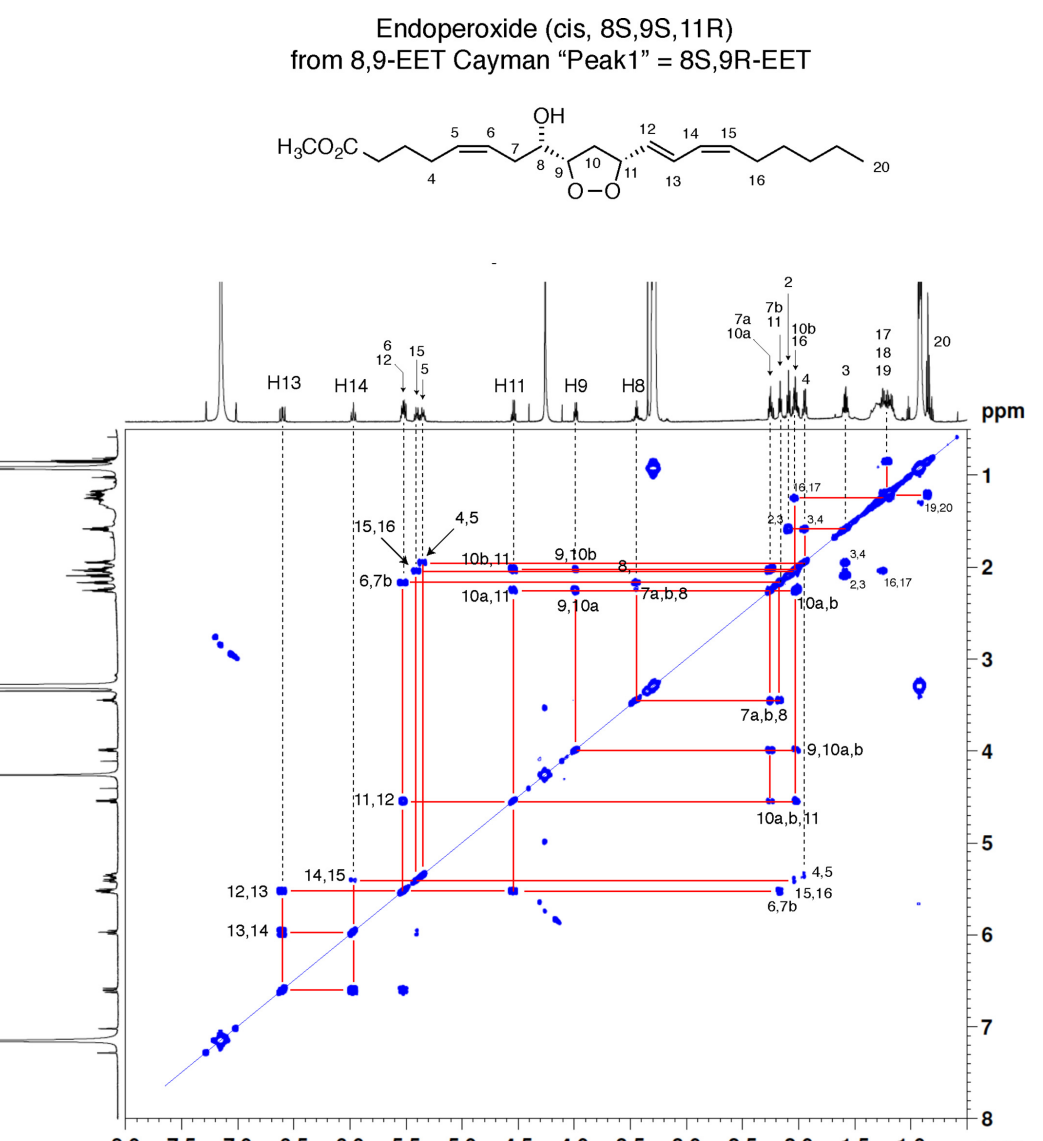
**^1^H NMR spectrum and COSY analysis of 8*S*-hydroxy-9*S*,11*R*-endoperoxy-5-*cis*,12-*trans*,14-*cis*-eicosatrienoate methyl ester in *d*_6_-benzene.**

**Figure 2.**
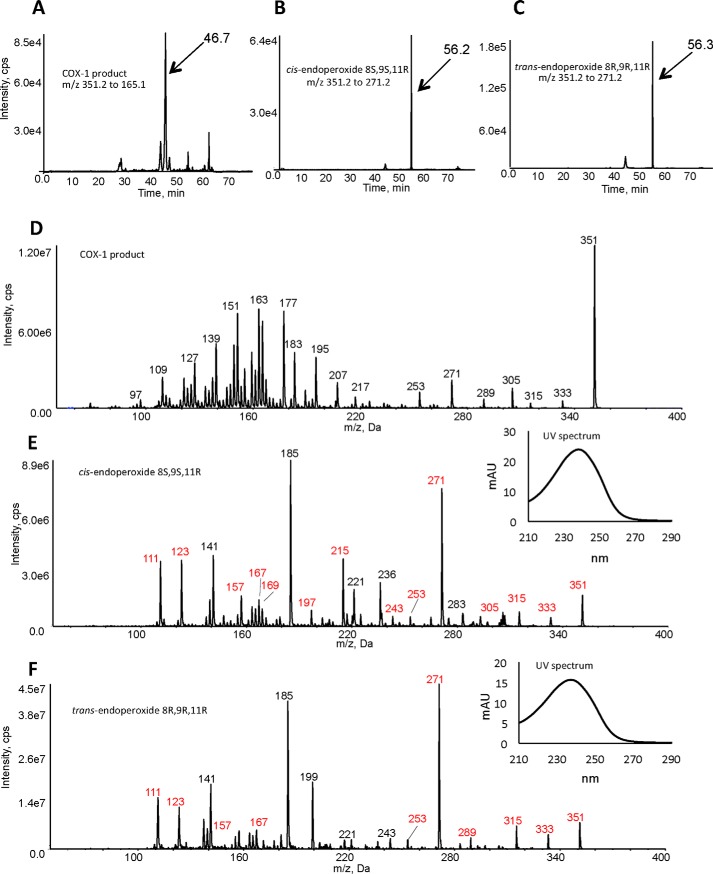
**Comparison of the COX-1–derived lipid with 8-hydroxy-9,11-dioxolanes indicates these are different lipids.** *A–C*, reverse-phase LC-MS/MS indicates that the lipids elute at different retention times. Lipids (COX-1 product or 8-hydroxy-9,11-dioxolanes) were separated using reverse-phase LC-MS/MS as described under “Experimental procedures” and compared for retention time. Lipids were detected either as *m*/*z* 351.2 → 165.1 (COX-1 lipid) or 351.2 → 271.1 (dioxolanes). *D--F*, comparison of MS/MS spectra from the COX-1–derived lipid *versus in vitro* generated dioxolanes along with confirmation of conjugated diene structures for dioxolanes. MS/MS spectra were acquired at the peak of elution for the lipids shown in *A–C* above. Ions shown in *red* for *E* and *F* are common to the COX-1 product. Nominal mass is shown as these are low-resolution spectra (tandem quadrupole mass spectrometer). The *inset* UV spectra for the dioxolanes were obtained by HPLC-UV analysis as indicated under “Experimental procedures.” *mAU*, milli-absorbance units.

First, we attempted to generate the proposed structure of DXA_3_, through oxidation of EETs using a recombinant LOX. In this way, two diastereomers were generated by oxidation of 8*S*,9*R*-EET and 8*R*,9*S*-EET using recombinant 8*R*-LOX (8), forming specifically the *cis*-endoperoxide product 8*S*,9*S*,11*R* (from 8*S*,9*R*-EET) and *trans*-endoperoxide product 8*R*,9*R*,11*R* (from the enantiomer 8*R*,9*S*-EET). ^1^H NMR confirmed the structures of these lipids, as shown for the *cis* 8*S*,9*S*,11*R*, and both showed expected conjugated diene UV spectra (Figs. 1 and 2). These were then compared using reverse-phase LC-MS/MS with the COX-1–derived lipid. However, they eluted later, and although their MS/MS spectra had some common fragment ions (labeled in *red*), they contained additional large ions that were absent in MS/MS spectra from the COX-1–derived lipid. In particular, these hydroxy-endoperoxides generated large daughter ions at *m*/*z* 185 quite distinct from the fragmentation properties of the platelet product (Fig. 2). Thus, these dioxolanes were inconsistent with the platelet lipid, and additional candidates were sought as described below.

### Scaling up generation of the “synthetic lipid” via arachidonate oxidation

We previously generated small amounts of the synthetic lipid by oxidation of 11-HpETE, as outlined by Porter and co-workers (6, 7), for synthesizing cholesteryl ester dioxolane derivatives. This lipid, which has the same MS/MS and reverse-phase retention time as the platelet isomer, was sufficient for MS/MS analysis, but the amounts produced were not enough to provide good NMR data.

**Figure 3.**
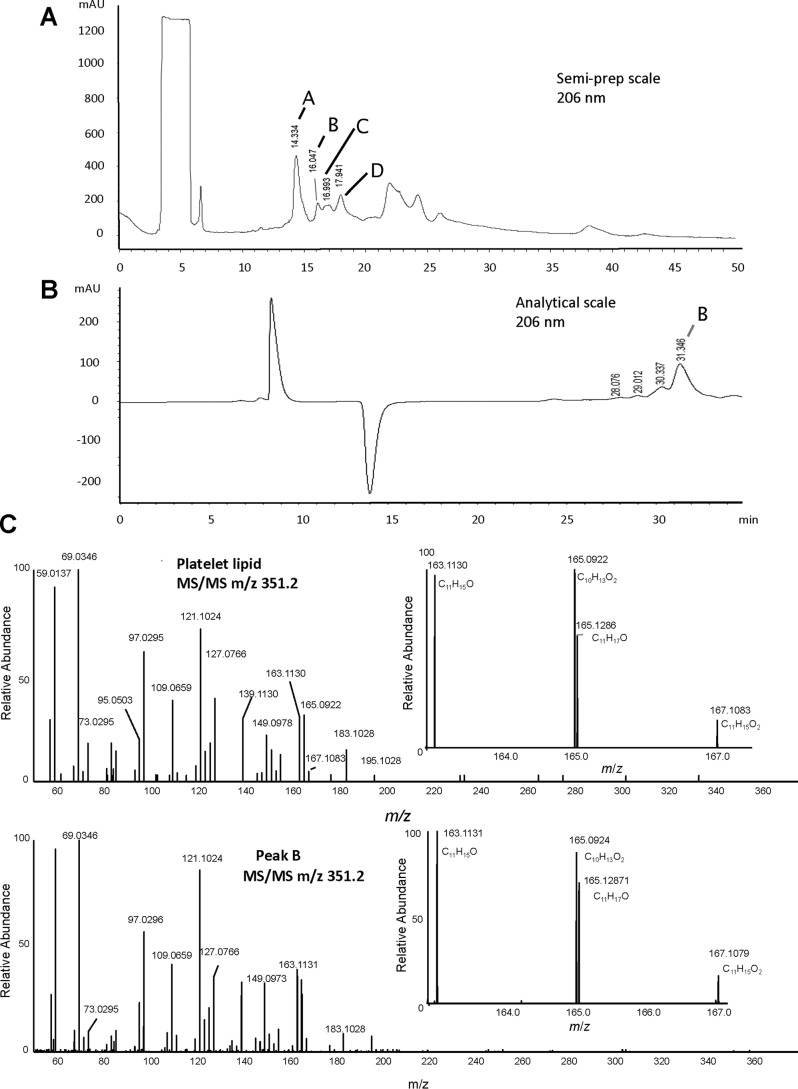
**Purification and high-resolution MS of lipids generated by oxidation of arachidonate to isolate the synthetic lipid.** *A*, semipreparative normal-phase HPLC as indicated in under “Experimental procedures” with collected fractions labeled *A–D*. The HPLC was monitored online at 206 nm. *B*, further HPLC purification of Peak B (Fig. 1*A*) as indicated under “Experimental procedures.” The HPLC was monitored online at 206 nm. *C*, high-resolution (Orbitrap) LC-MS/MS of platelet lipid (*top*) *versus* Peak B (*bottom*) as indicated under “Experimental procedures.” *mAU*, milli-absorbance units.

Herein, we modified this method based on observing that the synthetic lipid forms even during the oxidation of arachidonic acid while making 11-HpETE. In this way, the scaling up was simplified to a single process. Despite the fact that yields were quite low using this method (<0.05%), we managed to isolate the synthetic lipid in amounts (around 400 μg) that were enough for further analysis. Although large numbers of oxidized lipids are present in this crude reaction mixture, only one matched the platelet lipid's MS/MS spectrum and retention time (labeled Peak B in Fig. 3). A full description of the purification is presented under “Experimental procedures.”

It should be noted that all biologically active eicosanoids would be expected to form at low yield in a complex and uncontrolled free radical oxidation system such as this. If we consider a lipid such as PGE_2_, we expect that it would be present in such a reaction but at similarly low yield to what we found here, in addition to literally hundreds of other oxidation products.

**Figure 4.**
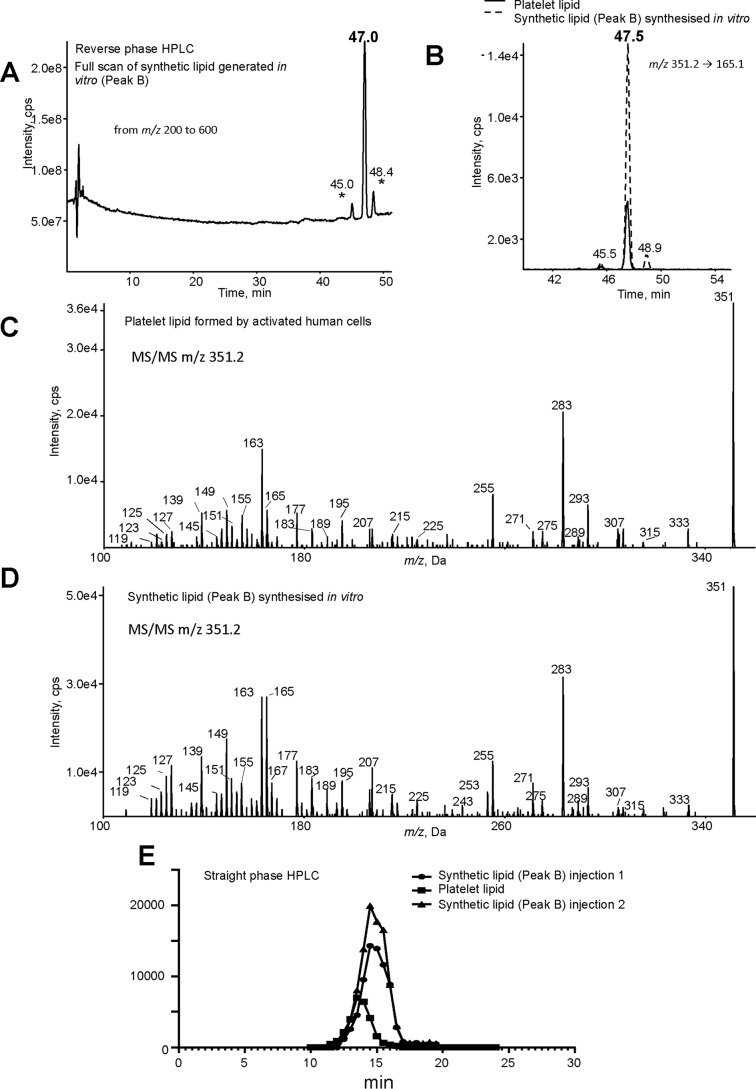
**Analysis of platelet lipid and Peak B confirms them to be isomers of the same lipid structure.** Washed human platelets were activated with 0.2 unit·ml^−1^ thrombin for 30 min at 37 °C, and lipids were extracted. Peak B was generated *in vitro* and purified as described under “Experimental procedures.” *A*, purified Peak B was analyzed using reverse-phase LC-MS/MS on the tandem quadrupole in full-scan mode. Full scan was carried out in negative mode, scanning Q3 from *m*/*z* 200 to 600. * shows the position of additional isomers of Peak B. *B*, reverse-phase LC-MS/MS of platelet lipid and Peak B showing coelution. Lipids were analyzed using reverse-phase LC-MS/MS, monitoring parent *m*/*z* 351.2 → 165.1. *C* and *D*, MS/MS spectra of the lipids obtained with a tandem quadrupole mass spectrometer. Lipids were separated as in *B*, and MS/MS spectra were acquired at the apex of elution using enhanced product-ion mode. Nominal mass is shown as these are low-resolution spectra. *E*, straight-phase HPLC of platelet lipid and Peak B shows the lipids separate. Lipids were resuspended in mobile phase and analyzed as outlined under “Experimental procedures.”

The lipid matching the platelet lipid based on MS/MS and retention time was targeted for further purification and characterization in this report as follows and is identified as the synthetic lipid previously characterized in our earlier study (4). Specifically, oxidized lipids were separated using normal-phase chromatography, with several products visible at 206 nm (Fig. 3*A*, labeled *A–D*). Fractions were collected and analyzed using high-resolution Orbitrap MS/MS and compared with platelet lipid (purified from activated platelets using reverse-phase LC). Peak B was found to have the same Orbitrap MS/MS spectrum and was further purified using reverse-phase HPLC and is thus identified as the synthetic lipid (Fig. 3, *B* and *C*). Synthetic lipid was next compared with the platelet lipid using a longer UPLC-MS/MS method for further structural confirmation. First, a Q1MS scan of the synthetic lipid shows a prominent peak around 47 min, indicating the most abundant component (Fig. 4*A*). Small peaks eluting immediately before and after are noted, as we previously published for the synthetic lipid generated using 11-HpETE oxidation, and are likely diastereomers (4). Next, both synthetic and platelet lipids were monitored using parent *m*/*z* 351.2 fragmenting to daughter ion *m*/*z* 165. Again, Peak B/synthetic lipid comprises one predominant isomer with two smaller peaks eluting immediately before and after, whereas the platelet lipid is a single peak (Fig. 4*B*). These three isomers in the synthetic lipid preparation have identical MS/MS spectra and likely represent quantitatively minor diastereomers of the platelet lipid as reported previously (data not shown) (4). Importantly, low-resolution MS/MS spectra acquired at the apex of the peak at 47.5 min using a tandem quadrupole instrument are virtually identical (Fig. 4, *C* and *D*). The synthetic and platelet lipids were next compared using straight-phase HPLC. Here, the synthetic lipid shows a slightly later retention time than the platelet isomer (Fig. 4*E*). This indicates that the lipids are likely different stereoisomers of the same compound. Peak B, as purified from Fig. 3*B*, was then taken forward for NMR analysis to inform us about the potential structure of the platelet lipid.

### NMR analysis of synthetic lipid identifies structure as 8,9–11,12-DiEp-13-HEDE

**Figure 5.**
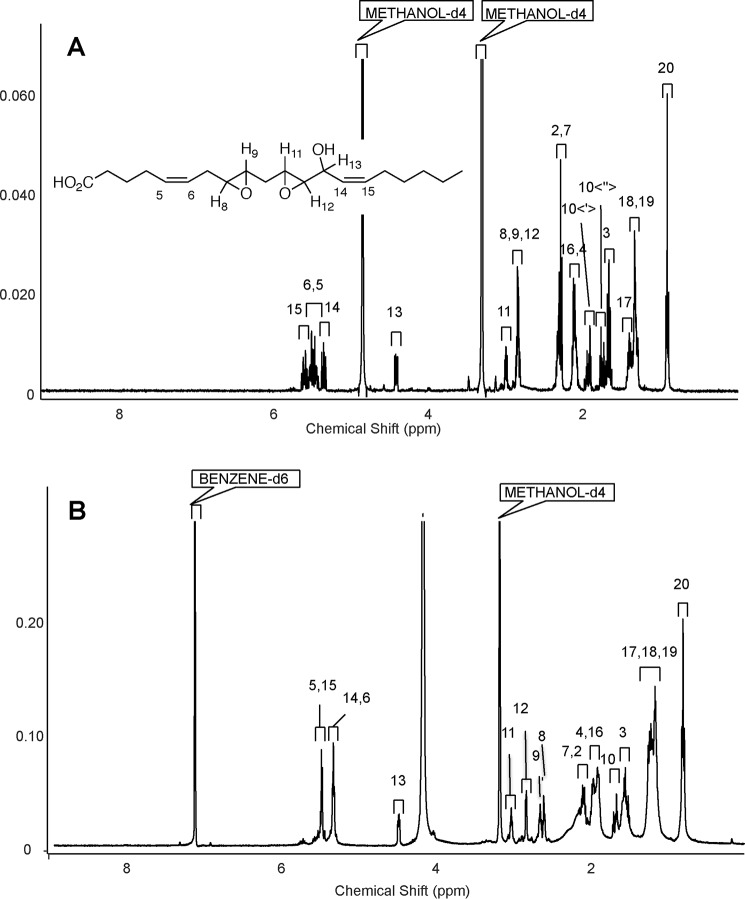
**^1^H NMR data of the synthetic lipid in methanol-*d*_4_ and benzene-*d*_6_ shows the structure as 8,9–11,12-DiEp-13-HEDE.** *A*, ^1^H NMR (methanol-*d*_4_; 400 MHz) δ 5.60 (dtd, 1H, *J* = 1.17, 7.54, 11.08 Hz), 5.48 (m, 2H), 5.35 (tdd, 1H, *J* = 1.56, 8.59, 10.94 Hz), 4.41 (ddd, 1H, *J* = 1.17, 4.10, 8.79 Hz), 2.97–3.00 (m, 1H), 2.86–2.81 (m, 3H), 2.34–2.31 (m, 2H), 2.28 (t, 2H, 7.23 Hz), 2.13–2.07 (m, 4H), 1.95–1.89 (m, 1H), 1.77–1.71 (m, 1H), 1.66 (qint, 2H, 7.23 Hz), 1.41–1.36 (m, 2H), 1,31–1.28 (br m, 4H), 0.90 (t, 3H, 7.0 Hz). *B*, ^1^H NMR (benzene-*d*_6_:methanol-*d*_4_, 90:10; 700 MHz) δ 5.51–5.43 (m, 2H), 5.36–5.29 (m, 2H), 4.48 (dd, 1H, *J* = 7.75, 4.0 Hz), 3.03 (dt, 1H, *J* = 2.20, 5.1 Hz), 2.83 (dd, 1H, *J* = 2.13, 3.83 Hz), 2.65 (dt, 1H, *J* = 2.25, 5.20 Hz), 2.61 (dt, 1H, *J* = 2.21, 5.36 Hz), 2.14–2.05 (m, 2H), 2.02–1.85 (m, 4H), 1.72–1.63 (dt, 1H, *J* = 15.03, 4.58 Hz), 1.58–1.48 (m, 3H), 1.31–1.10 (m, 8H), 0.81 (t, 3H, *J* = 6.9 Hz).

**Figure 6.**
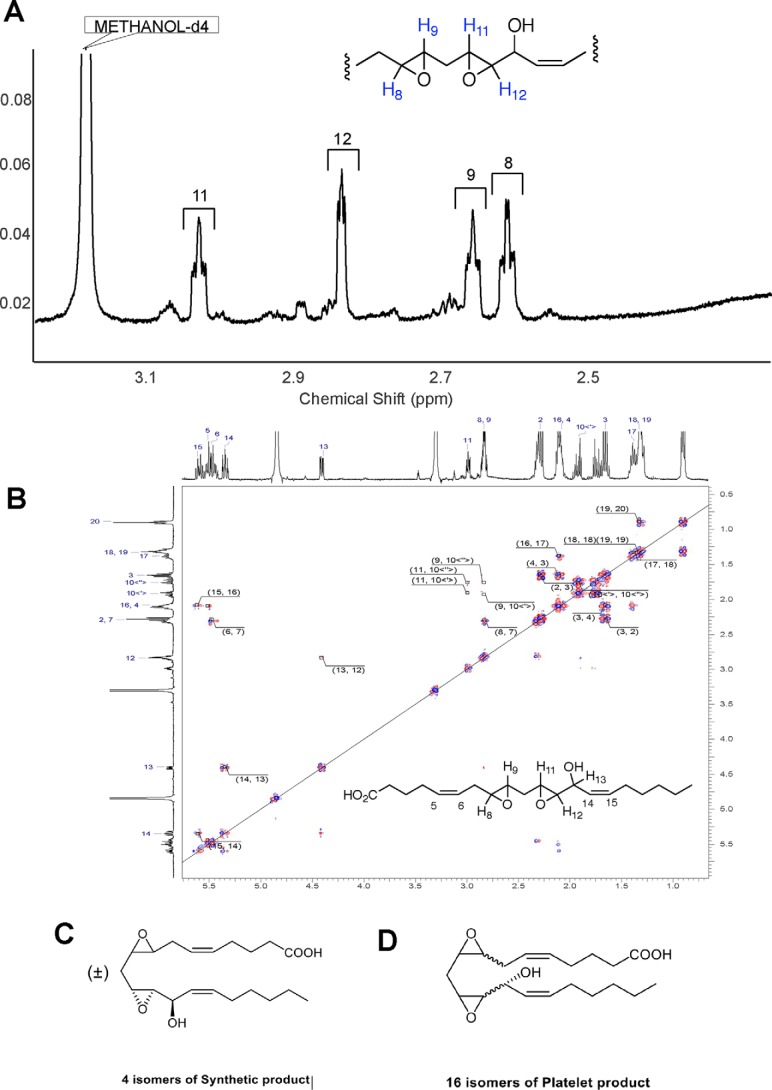
**^1^H NMR data of the synthetic lipid continued.** *A*, region of epoxy protons ^1^H NMR (benzene-*d*_6_:methanol-*d*_4_, 90:10; 700 MHz) δ 3.04–3.01 (dt, 1H, *J* = 2.20, 5.1 Hz), 2.84–2.83 (dd, 1H, *J* = 2.13, 3.83 Hz), 2.66–2.64 (dt, 1H, *J* = 2.25, 5.20 Hz), 2.62–2.60 (dt, 1H, *J* = 2.21, 5.36 Hz). *B*, 2D COSY. *C*, structure of the synthetic generated diEHEDE isomer (±)8,9-*trans*-11(*R*),12(*R*)-*trans*-DiEp-13(*R*)-HEDE. *D*, structure of the proposed platelet lipid 8,9–11,12-DiEp-13-HEDE. Shown here is the isomer proposed based on studies using mutant COX-2 isoforms, 8(*R*),9(*R*)-*cis*-11(*R*),12(*R*)-*trans*-diEp-13(*R*)-HEDE.

Structure elucidation of synthetic lipid as 8,9–11,12-DiEp-13-HEDE was achieved via ^1^H NMR and COSY. For structure numeration and assignment, refer to Figs. 5, *A* and *B*, and 6*A* and Table 1. Preliminary determination began with the identification of H13, which was assigned based on the ^1^H NMR of a similar compound obtained by Brash and co-workers (9). Upon identification of the H13 proton, assignment of the epoxide and olefinic protons was possible based on ^1^H-^1^H correlations acquired from the COSY spectrum shown in Fig. 6*B* and Table 1. Correlations are observed from H13 to the H14 olefinic proton and to the epoxide proton H12, allowing assignment of these protons. H14 is correlated with the most downfield olefinic proton, which was assigned as H15, and the olefin ^1^H-^1^H coupling of 11 Hz confirms that this double bond has the *Z* configuration (Fig. 5*A*). This allows the assignment of the peaks between H14 and H15 as the two other olefinic protons. However, protons H5 and H6 are unassigned because their signals appear as multiplets (Fig. 5*A*). Given the structure of the AA substrate used to generate the synthetic lipid, we propose *cis* geometry for the double bond at 5,6.

**Table 1:** **COSY assignment of 8,9–11,12-DiEp-13-HEDE using ^1^H-^1^H gradient double quantum COSY (methanol-d_4_, 400 MHz)**

F2 axis	F1 axis	Correlation
*ppm*	*ppm*	
1.33	0.89	H20–19
1.38	1.33	H18–17
1.65	2.29	H3–2
1.66	2.08	H4–3
1.92	1.76	H10′-10″
2.1	1.38	H17–16
2.83	2.29	H8–7
2.84	1.93	H10′-9
2.84	1.76	H10″-9
3.00	1.90	H11–10′
3.00	1.76	H11–10″
4.41	2.84	H13–12
5.35	4.39	H14–13
5.48	2.29	H7–6
5.51	2.09	H5–4
5.61	5.35	H15–14
5.62	2.08	H16–15

These protons are coupled to methylene CH_2_ protons H4 and H7 (Fig. 6*B* and Table 1). H13 shows correlation to H12 (integrating at ∼3.0) at 2.83 ppm, which leaves the single proton at 2.99 ppm to be H11 or H9 (Fig. 6*B* and Table 1). Correlation from the H10 protons to two epoxide protons suggests the final structure as the methylene-bridged diepoxide shown (Fig. 6, *B* and *C*, and Table 1). In summary, we propose that both epoxy rings are located in positions 8,9 and 11,12 where the double bonds of AA were originally sited.

Although assignment of the ^1^H NMR and the COSY in CD_3_OD allowed us to obtain a general structure, we were interested in gathering additional structural information about the relative stereochemistry of some functional groups in our molecule as was done previously (10). Initial attempts to assign relative stereochemistry between the hydroxyl and 11,12-epoxide via measurement of *J* couplings were unsuccessful due to the overlap of epoxide protons. Although the synthetic lipid was completely insoluble in benzene, a 90:10 mixture of benzene-*d*_6_:methanol-*d*_4_ was found to yield a proton spectrum in which these epoxide protons were completely separated (Figs. 5*B* and 6*A*). Here, we acquired a proton spectrum that resolved the assignment and *J* couplings for these epoxide protons. As shown in Fig. 5*B*, protons in positions 8 and 9 as well as 11 and 12 contained a small coupling constant in the range of 2.1–2.3 Hz, which is consistent with *trans* configuration of epoxy rings (9). Thus, on the basis of the 2-Hz coupling constants, the NMR analysis defines both the 8,9- and 11,12-epoxide groups as *trans*-epoxy, but their absolute stereochemistry has not been established.

To summarize our NMR data, the synthetic lipid is shown to be 8,9–11,12-diepoxy-13-hydroxyeicosadienoic acid using NMR and MS/MS. Furthermore, NMR proves the position of the double bond at 14,15 with *cis* geometry. NMR also proves that the relative configuration of the hydroxyl group at 13 and the adjacent carbon atom of the epoxy ring at 11,12 is *trans*. Overlapping multiplets for H5 and H6 prevented unambiguous assignment of this double bond configuration. However, based on the structure of AA, we propose that the double bond located at 5,6 has *cis* geometry. Both epoxy rings are confirmed as *trans* configuration. We cannot determine the absolute configuration of the hydroxyl group and both epoxy rings from our NMR data. However, the epoxides will be either *S*,*S* or *R*,*R*, whereas the hydroxyl will be the same configuration as the epoxy ring at position 11,12. Thus, the list of likely structures for the synthetic lipid includes 8*R*,9*R-trans*-11*R*,12*R-trans*-DiEp-13*R*-HEDE, 8*S*,9*S-trans*-11*R*, 12*R-trans*-DiEp-13*R*-HEDE, 8*R*,9*R-trans*-11*S*,12*S-trans*-DiEp-13*S*-HEDE, and 8*S*,9*S-trans*-11*S*,12*S-trans*-DiEp-13*S*-HEDE with both double bonds predicted as *cis*. With the amount of lipid we were able to generate using our current synthetic approach, NMR studies are unable to distinguish the absolute configuration further than this. The proposed structure is shown as Fig. 6*C* and is also informed by the data in Schneider *et al.* (9) in which the initial epoxide relative stereochemistry is set via the 9,11-endoperoxide intermediate.

### Proposed MS/MS fragmentation for DiEpHEDE

Overall, our new data showing an identical MS/MS spectrum and reverse-phase retention time to the synthetic lipid generated here confirm the platelet lipid to be 8,9–11,12-DiEp-13-HEDE, whereas the different chiral LC retention time indicated that the synthetic and platelet lipids are different isomers. We next considered which candidate isomers are most likely for the platelet lipid. Given the presence of *cis* double bonds in AA and that COX enzymes generate 13*R*-hydroperoxyl/hydroxyl isomers during turnover, we predict that the platelet lipid forms in line with these structural features. Given this, we propose that there are 16 potential isomers for the platelet lipid. This is based on the potential for *cis*- or *trans*-epoxides and resulting stereochemical configurations possible (Fig. 6*D*). Unfortunately, a total synthetic approach to generate all 16 would take several years and is beyond the scope of this current study. We note that Schneider *et al*. (9) previously showed a *cis*-epoxide at 9,10 and *trans*-epoxide at 11,12 for an 8,9–11,12-DiEp-13-HEDE isomer generated by a mutant COX-2 isoform; however, without further evidence for this configuration in the platelet lipid we have not assigned this geometry (see “Discussion” for more detail on this).

Next, based on our findings, the MS/MS fragmentation route previously presented for the platelet lipid based on extensive high-resolution MS/MS of native and deuterated analogs and MS^3^ was re-evaluated. Most daughter ions mapped to similar fragmentation routes, and all major ions could be assigned to this the structure, including *m*/*z* 195, which could not previously be accounted for (Schemes S1–S5). Possible mechanisms involved in the formation of *m*/*z* 255, 207, and 163 are presented in Scheme S1 as well as mechanisms for *m*/*z* 333 and 289 (Scheme S2), *m*/*z* 183 and 139 (Scheme S3), *m*/*z* 195 and 177 (Scheme S4), and *m*/*z* 315 and 271 (Scheme S5). All of the proposed reaction pathways taking place in the gas phase after collisional activation have been described previously for epoxy fatty acids, and proposed fragment ions are consistent with the high-resolution mass spectrometric measurements and deuterium-labeled analog published previously (4, 10).

### 8,9–11,12-DiEp-13-HEDE activates leukocyte integrin expression

**Figure 7.**
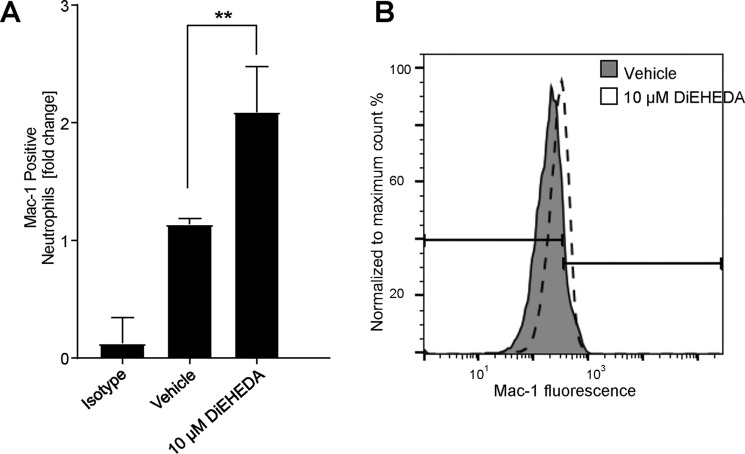
**DiEpHEDE activates neutrophil Mac-1 expression.** Neutrophils were incubated with synthetic DiEpHEDE (*diEHEDA*) or vehicle for 20 min at 37 °C, and Mac-1 expression was monitored by flow cytometry using anti-human CD11b (Mac-1)-Alexa Fluor 647. Where stated, neutrophils were preincubated with 10 μm synthetic DiEpHEDE or vehicle for 10 min at 37 °C and then activated with 10 μm fMLP for a further 10 min at 37 °C. *A*, bar chart showing activation of Mac-1 expression by DiEpHEDE. Data represent -fold change in fluorescence signal normalized to vehicle control (*n* = 3, mean ± S.E. (*error bars*)). Statistical significance used one-way analysis of variance followed by Bonferroni post hoc test: *, *p* < 0.05. *B*, representative histogram depicting increased Mac-1 expression following activation with DiEpHEDE. The *line* represents the Mac-1–positive neutrophil gate as set using untreated neutrophils.

We previously found that a semipurified platelet-derived DiEpHEDE isomer can activate neutrophil integrin expression (4). To investigate whether synthetic 8,9–11,12-DiEp-13-HEDE retained the same property as the platelet isomer, we repeated the experiment on three separate donor isolates as shown using the synthetic lipid (Fig. 7, *A* and *B*). In our earlier study, we also found that DiEpHEDE can stimulate priming of neutrophils at far lower amounts of 10 nm. Herein, we repeated this several times, but it was not consistent in all donor isolations, only being observed in around 50%. This suggests reduced potency of the synthetic lipid and is also consistent with it being a different isomer.

## Discussion

We recently identified a new lipid generated by COX-1 in human platelets that can activate neutrophils and proposed the structure as 8-hydroxy-9,11-dioxolane eicosatetraenoic acid based on extensive biochemical and chemical evidence but without NMR (4). We now present ^1^H and COSY NMR data, which revise the structure to 8,9–11,12-DiEp-13-HEDE, a metabolite that we suggest forms in platelets through opening of an initially formed dioxolane ring followed by epoxidation, oxygen insertion, and reduction as explained in detail below (Fig. 6*D*). Based both on the biology of COX-1 and structure of the substrate AA, we propose that the primary synthetic lipid is one of four potential isomers, whereas the platelet lipid is one of 16, noting that the platelet isomer is not present in the synthetic lipid preparation. This is evidenced by the two lipids having different retention times on chiral-phase chromatography (Fig. 4*E*).

Herein, we tested the ability of the synthetic lipid to either activate (directly) or prime neutrophils in response to the bacterial peptide fMLP for Mac-1 activation. In the case of priming, the lipid is added first at far lower concentrations, and then the ability of fMLP to activate is tested following a preincubation step. In our previous study, we showed that the platelet lipid could activate directly at 10 μm, and it also primed at 10 nm (4). Here, the synthetic lipid showed an ability to activate neutrophils; however, for priming it was less effective than we observed previously with the platelet lipid. It is well-known that in terms of lipid signaling, isomeric structure is critical to biological function (*e.g.* recognition by G protein–coupled receptors). This is similar to prostaglandins and their enantiomers (isoprostanes), which show similarities but also reduced potency in signaling ability Thus, the synthetic lipid may be less bioactive, leading it to behave differently from the platelet isomer in bioassays at extremely low concentrations. We expect that the amounts of DiEpHEDE generated by platelets will be sufficiently high for signaling at low concentrations. Specifically, we detected generation of around 6 ng/10^8^ cells, while at the same time there was 1.6 ng of PGE_2_ and 25 ng of thromboxane B_2_ (4). Importantly, despite lower amounts of PGE_2_, this lipid is well-known to be bioactive toward platelets (11). This indicates that a platelet eicosanoid generated in lower amounts than DiEpHEDE is already known to be bioactive, providing confidence that DiEpHEDE is generated in biologically relevant amounts while noting that generation of this lipid in platelets is sensitive to aspirin (4). Although we have not tested the mechanism of action, activation of leukotriene B4 (BLT) receptors that are activated by leukotrienes is one possibility. Lipids generated by vascular cells can possess multiple bioactivities, and an exhaustive examination (*e.g.* vasoconstriction, activation of platelets, leukocytes to express integrins, degranulation, mobilization of calcium, shape change, phagocytosis, aggregation, etc.) would be out of the scope of this study.

**Figure 8.**
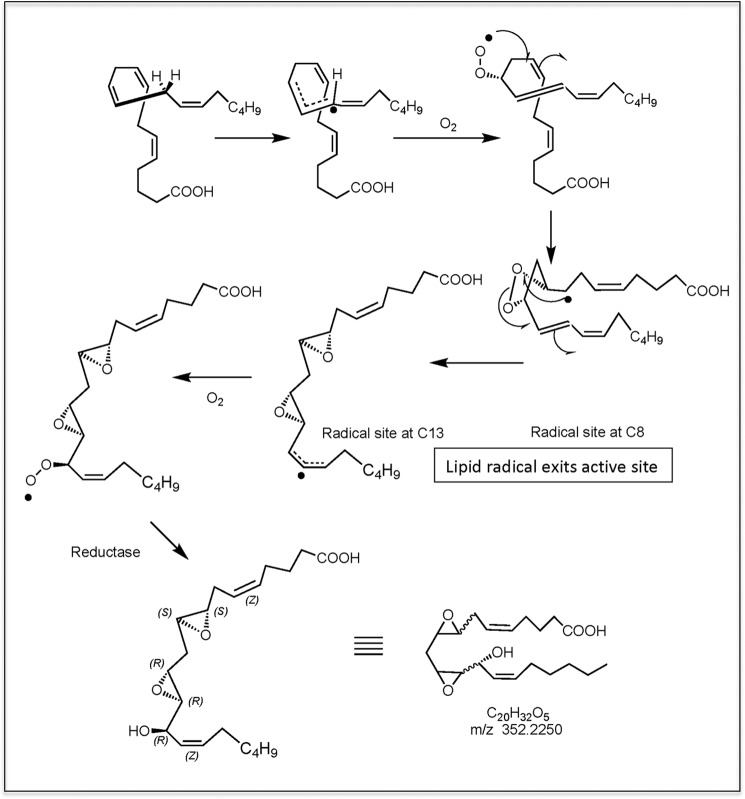
**Proposed route to synthesis of 8,9–11,12-DiEp-13-HEDE by COX isoforms.**

Previously, we demonstrated that the platelet lipid can be synthesized by COX-1 in intact cells or by recombinant COX-1 or -2 *in vitro* (2). A mechanism was proposed whereby a 9,11-dioxolane lipid with carbon-centered radical at C8 exited the active site and then reacted with O_2_ before reduction to form the product. In those studies, we established that the dioxolane ring forms before lipid exits the active site using mutant COX-2 enzymes; thus, using this information, we now present a revised route to formation of 8,9–11,12-DiEp-13-HEDE taking this into account as follows (Scheme S1). (i) 9,11-Dioxolane with carbon-centered radical at C8 exits the active site. (ii) Intramolecular attack by the C8 radical on the dioxolane leads to ring opening and reaction by the oxygens on C8 and C12, forming diepoxides and leaving a carbon-centered radical at C13. This then reacts with O_2_ before reduction to the 13-hydroxy lipid. Although the mechanism in Fig. 8 suggests that the lipid radical exits the active site, another possibility is that the second diatomic oxygen attacks while in the active site, similar to the mechanism by which the endoperoxide PGG_2_ is formed in the cyclooxygenase pathway. The platelet lipid appears as a single isomer on chiral- and reverse-phase HPLC, which means that the formation of this lipid is stereochemically controlled. This led us to propose that the dioxolane is formed prior to radical exit.

Formation of diepoxyeicosanoids by COX-2 isoforms has been reported previously (9). Thus, we had previously considered these as potential candidates for the platelet lipid. However, they were discounted based on several lines of evidence, in favor of the dioxolane at that time, as follows.

1) Only some mutant COX-2 isoforms have been shown to generate diepoxyeicosanoids, including 8,9–11,12-DiEp-13-HEDE. In previous studies, both WT COX-2 and the W387F mutant used herein were unable to make the diepoxide lipid (4, 9). As this contrasted with our findings where both could make this new lipid, we considered that specific mutations to the active site are required to enable COX isoforms to facilitate the exit of the dioxolane followed by ring opening and epoxidation. We consider that one potential explanation for our findings was the use of different extraction conditions: in this study, we may have favored extraction of more polar products such as these than in the previous work.

2) During purification of the platelet lipid, a lipid with absorbance suggestive of the presence of a conjugated diene chromophore eluted from the HPLC column (Fig. 3, *A* and *B*, in Ref. 4). This was consistent with the proposed structure of “DXA_3_” but not with diepoxides. LC/UV analysis of the purified concentrated DiEpHEDE undertaken for this study confirms that no UV-active chromophores are present (data not shown). Thus, we have now revisited our raw data from the previous study and determined that the absorbance most likely originated from a contaminating lipid, also made by platelets, present in the preparation eluting at the same time. Specifically, using MS data from biogenic standards we had available at the time of the previous study, we can estimate that the amount of DiEpHEDE eluting in the peak represents around 3.5 μg. However, based on absorbance, the conjugated diene represents only 70 ng of product. Thus, given the amount of platelet lipid eluting, this UV spectrum is too weak to account for the structure proposed.

3) Dioxolane lipids were previously synthesized by Porter and co-workers (6, 7) via oxidation of cholesteryl 11-HpETE. In our first study, we repeated this reaction using the free acid 11-HpETE as substrate and detected significant amounts of the lipid (4). This provided proof of concept that the 11-hydroperoxyl radical of AA can serve as a precursor for the platelet lipid under conditions where dioxolanes are known to form *in vitro*. This was consistent with our proposed mechanism of formation via COX isoforms.

Aside from the UV spectrum presented previously (addressed in point 2 above), all our structural characterization data previously published are directly applicable to the new structure and support the assignment (4). These extensive data include derivatization and GC-MS showing one hydroxyl group, no carbonyls, four rings/double bonds, and two oxygens added to arachidonate (4). Our previous MS/MS and MS^3^ data, including stable isotope labeling studies, are reinterpreted to provide new fragmentation pathways, which are fully consistent with the structure and synthesis pathway (Fig. 6*D* and Schemes S1–S5). The corrected structure, confirmed to be generated by thrombin-activated human platelets by COX-1 and to activate leukocyte integrin expression *in vitro*, is assigned to 8,9–11,12-DiEp-13-HEDE.

## Experimental procedures

HPLC-grade solvents were from Thermo Fisher Scientific (Hemel Hempstead, Hertfordshire, UK). Anti-human CD11b-Alexa Fluor 647 was from BioLegend. All other reagents were from Sigma unless otherwise stated.

### Synthesis of platelet lipid

Human blood donations were approved by the Cardiff University School of Medicine Ethics Committee and were obtained with informed consent (SMREC 12/37 and SMREC 12/10) and according to the Declaration of Helsinki. For studies on isolated platelets, whole blood was collected from healthy volunteers free from nonsteroidal anti-inflammatory drugs for at least 14 days into acid–citrate–dextrose (ACD; 85 mmol/liter trisodium citrate, 65 mmol/liter citric acid, 100 mmol/liter glucose) (blood:ACD, 8.1:1.9, v/v) and centrifuged at 250 × *g* for 10 min at room temperature. Platelet-rich plasma was collected and centrifuged at 900 × *g* for 10 min, and the pellet was resuspended in Tyrode's buffer (134 mmol/liter NaCl, 12 mmol/liter NaHCO_3_, 2.9 mmol/liter KCl, 0.34 mmol/liter Na_2_HPO_4_, 1.0 mmol/liter MgCl_2_,10 mmol/liter HEPEs, 5 mmol/liter glucose, pH 7.4) containing ACD (9:1, v/v). Platelets were centrifuged at 800 × *g* for 10 min and then resuspended in Tyrode's buffer at 2 × 10^8^ ml^−1^. Platelets were activated at 37 °C in the presence of 1 mmol/liter CaCl_2_ for 30 min with 0.2 unit·ml^−1^ thrombin before lipid extraction.

### Platelet lipid extraction

Lipids were extracted by adding a solvent mixture (1 m acetic acid:2-propanol:hexane, 2:20:30) to the sample at a ratio of 2.5 ml of solvent mixture to 1 ml of sample, vortexing, and then adding 2.5 ml of hexane. Following vortexing and centrifugation (300 × *g*, 5 min), lipids were recovered in the upper hexane layer. The samples were then re-extracted by the addition of an equal volume of hexane followed by further vortexing and centrifugation. The combined hexane layers were then evaporated to dryness using a Rapidvap N2/48 evaporation system (Labconco Corporation) and resuspended in 200 μl of MeOH. Platelet lipid extract was stored at −80 °C until LC-MS/MS analysis.

### Synthesis and purification of lipid matching COX-1 metabolite from platelets

Arachidonic acid was mixed with α-tocopherol (5% by weight) in a round-bottom flask, and oxygen was bubbled through this mixture under stirring at 37 °C for 3 days. The resulting mixture was purified using flash chromatography on silica gel (hexane:ethyl acetate:acetic acid, 90:10:0.5–50:50:0.5), and fractions were analyzed using high-resolution LC-MS/MS (see Fig. 3). Lipids were purified using isocratic separation with a mobile phase of 95:5:0.1 heptane:isopropyl alcohol:acetic acid on a Luna semipreparative silica (5 μm) 250 × 10-mm column at 5 ml/min, monitoring at 206 nm (Fig. 3*A*). A second clean-up step utilized an isocratic separation of 97:2.5:0.5:0.1 heptane:isopropyl alcohol:methanol:acetic acid with a Luna silica (5 μm) 250 × 4.6-mm column at 0.5 ml/min, monitoring at 206 nm (Fig. 3*B*).

### Synthesis and purification of 8-hydroxy-9,11-dioxolane eicosatetraenoic acid isomers by LOX oxidation of 8,9-epoxyeicosatetraenoic acids

(±)8,9-EET (Cayman Chemical) was resolved by semipreparative chiral HPLC (12), and the enantiomers were each incubated with recombinant *Plexaura homomalla* 8*R*-lipoxygenase similarly to other lipoxygenase reactions with EETs described previously by Teder *et al.* (13) Briefly, 8,9-EET (50 μm) was incubated in 0.1 m phosphate, pH 8, with 8*R*-LOX, and the progress was followed by UV scanning (200–350 nm), with appearance of a conjugated diene chromophore with λ_max_ around 240 nm (see Ref. 13). Upon complete of the reaction, the products were extracted using a 30-mg Oasis cartridge (Waters), eluted with methanol, and subsequently purified by reversed-phase and normal-phase HPLC. The two main products are an 11*R*-hydroperoxide derivative with the 8,9-epoxide moiety still intact (8,9-epoxy-11*R*-hydroxy-5*Z*,12*E*,14*Z*-eicosatrienoic acid) and the target compound 8-hydroxy-9,11-endoperoxy-5*Z*,12*E*,14*Z*-eicosatrienoic acid. The hydroxy-endoperoxide isomers were generated from both 8*R*,9*S*- and 8*S*,9*R*-EET enantiomers and purified as the methyl ester derivatives for assignment by ^1^H NMR. Subsequently, the free acids were isolated and compared by LC-MS with the bioactive platelet product. (For comparison of ^1^H NMR and LC data, an equivalent set of derivatives was also prepared by 8*R*-LOX reactions with 5,6-EET, providing 5-hydroxy-6,8-endoperoxy-9*E*,11*Z*,14*Z*-eicosatrienoic acids; these aided in assigning the mass spectral fragmentations of the target hydroxy-endoperoxides derived from 8,9-EET). NMR data on these lipids are provided in Fig. S1.

### NMR methods

A Varian Unity Inova 400- or 700-MHz spectrometer was used for NMR of diepoxy compounds in CD_3_OD or *d*_6_-benzene. Postacquisition processing was performed in ACD/Spectrus processor 2017.2. ^1^H NMR and ^1^H-^1^H COSY NMR spectra on the hydroxy-endoperoxides were obtained on a Bruker 600-MHz spectrometer at 298 K. The ppm values are reported relative to residual nondeuterated benzene (C6D6; δ = 7.15 ppm) All spectra were analyzed on Bruker TopSpin 3.0 software.

### Straight-phase HPLC

Lipids were dried and then resuspended in mobile phase (hexane:2-propanol:acetic acid, 90:10:0.1) and injected onto a Chiralcel OD 250 × 4.6-mm column (Chiral Technologies Ltd., Exton, PA) with isocratic separation at 1 ml·min^−1^. Fractions were collected at 30-s intervals and analyzed by direct-flow injection monitoring *m*/*z* 351.2 → 165.1 on a tandem quadrupole ion trap mass spectrometer (4000 qTrap, Sciex, Thornhill, Canada).

### Reverse-phase LC-MS/MS

LC-MS/MS analysis of human platelet-derived and synthetic DiEpHEDE was performed on a Nexera LC system (Shimadzu) coupled by electrospray ionization to a tandem quadrupole ion trap mass spectrometer (4000 qTrap). Briefly, LC was performed at 40 °C using a C_18_ Spherisorb ODS2 (5 μm) 150 × 4.6-mm column (Waters) at a flow rate of 1 ml·min^−1^ over 75 min. Mobile phase A was water, 0.1% formic acid, and mobile phase B was acetonitrile, 0.1% formic acid. The following linear gradient for B was applied: 20% for 0.5 min, 20–42.5% over 50 min, 42.5–90% from 50 to 60 min, and held at 90% for 5 min followed by 10 min at initial condition for column re-equilibration. Injection volume was 10 μl. Ionization was performed using electrospray ionization in the negative mode (ESI−), monitoring parent ion to daughter ion *m*/*z* 351.2 → 165.1 (dwell time, 200 ms) with the following parameters: TEM, 650 °C; GS1, 70 p.s.i.; GS2, 55 p.s.i.; CUR, 40 p.s.i.; ESI spray voltage, −4.3 kV; DP, −53 V; EP, −10 V; CE, −26 V; and CXP at −7 V. Full-scan MS was carried out in negative mode, scanning Q3 from *m*/*z* 200 to 600 with total scan time (including pauses) over 4 s. Settings were: TEM, 650 °C; GS1, 60 p.s.i.; GS2, 30 p.s.i.; CUR, 35 p.s.i.; ESI spray voltage, −4.5 kV; DP, −55 V; EP, −10 V; CXP at −30 V; and IHE on.

### High-resolution LC-MS/MS

High-resolution LC-MS/MS analysis was carried out on an Ultimate 3000 UPLC (Thermo Scientific) coupled to a Q Exactive mass spectrometer (Thermo Scientific). Mass accuracy was determined to be better than 1 ppm. Liquid chromatography was performed using Phenomenex Kinetex C_18_, 2.6 μm, 100 × 2.1 mm at a flow rate of 400 μl·min^−1^ over 25 min. Mobile phases A and B were identical to the method above but with a gradient for B of 15–28% over 0–1 min, 28–42% over 1–4 min, 42–60% over 4–6 min, 60–80% over 6–16 min, 80–95% over 16–19 min, and held at 95% over 19–22 min followed by 3-min re-equilibration to 15%. Injection volume was 10 μl. Electrospray ionization was used in negative mode with a sheath gas flow rate of 60 units and capillary temperature at 320 °C. Data-dependent MS/MS of *m*/*z* 351 was carried out with a resolving power of 35,000.

### HPLC-UV analysis of COX-1–derived lipid

Arachidonic acid was oxidized using recombinant ovine COX-1 *in vitro*. The enzyme was incubated with 2 molar eq of hematin in 100 mm phosphate buffer for 20 min on ice. 3.4 μg of COX-1 was incubated with 150 μm arachidonate and 500 μm phenol in 100 mm phosphate buffer for 3 min at 37 °C under oxygen atmosphere. Lipids were extracted using the hexane–isopropyl alcohol extraction described above. Lipids from multiple COX-1 reactions were combined, and DiEpHEDE was HPLC-purified employing a Spherisorb ODS2 column (5 μm, 150 × 4.6 mm; Waters) with LC settings and mobile phases as described under “Reverse-phase LC-MS/MS.” Fractions were collected every 30 s, and DiEpHEDE was identified by MS/MS, monitoring *m*/*z* 351.2 → 165.1. DiEpHEDE was extracted using solid-phase extraction using silica-based solid-phase extraction columns (Sep-Pak, Waters) that were equilibrated with 5 ml of water, pH 3.0, followed by 5 ml of methanol. The DiEpHEDE sample was diluted with water to an organic content of 15% and purified over the column. The column was washed with 20 ml of water at pH 3.0, and the lipid was extracted using 5 ml of methanol. Extracts were dried using nitrogen flow, and the lipid was dissolved in methanol. About 3.5 μg of DiEpHEDE was injected on an LC/UV system (Infinity II, Agilent) using the same LC settings, and absorbance was measured at 235 nm.

### Isolation and activation of human neutrophils

Human neutrophils were isolated from 20 ml of citrate-anticoagulated whole blood and resuspended in Krebs buffer (100 mmol/liter NaCl, 50 mmol/liter HEPES, 5 mmol/liter KCl, 1 mmol/liter NaH_2_PO_4_, 2 mmol/liter d-glucose, 1.25 mmol/liter MgCl_2_, 2 mmol/liter CaCl_2_, pH 7.4). Briefly, blood was mixed 1:3 with 2% trisodium citrate (w/v) and HetaSep (STEMCELL Technologies) and allowed to sediment for 45 min at 20 °C. The upper plasma layer was recovered and underlaid with ice-cold Lymphoprep (2:1 plasma:Lymphoprep) and centrifuged at 800 × *g* for 20 min at 4 °C. The pellet was resuspended in ice-cold PBS and 0.4% sodium tricitrate (w/v) and centrifuged at 400 × *g* for 5 min at 4 °C. Contaminating erythrocytes were removed using up to three cycles of hypotonic lysis. Finally, cells were resuspended in a small volume of Krebs buffer, counted, and kept on ice. Neutrophils were diluted to 2 × 10^6^ cells/ml and incubated in the presence of DiEHEDE or vehicle for 20 min at 37 °C. In some experiments, neutrophils were preincubated with 10 nm DiEpHEDE or vehicle for 10 min at 37 °C and then activated with 10 μm fMLP for a further 10 min at 37 °C. Nonspecific antibody binding was blocked using 5% mouse serum in ice-cold FACS buffer (PBS containing 0.5% bovine serum albumin (BSA), 5 mmol/liter EDTA, 2 mmol/liter sodium azide, pH 7.4) for 1 h on ice and centrifuged at 320 × *g* for 5 min at 4 °C. Cells were washed with ice-cold FACS buffer, and anti-human CD11b-Alexa Fluor 647 or isotype control was added and incubated for 30 min on ice (4). Neutrophils were washed twice with ice-cold FACS buffer and analyzed with a FACSCanto II flow cytometer (BD Biosciences) and FlowJo software.

### Statistics

Data are expressed as mean ± S.E. of three separate determinations. Statistical significance was assessed using one-way analysis of variance followed by a Bonferroni multiple-comparisons test with Prism software version 7 (GraphPad). *p* < 0.05 was considered statistically significant.

## Author contributions

A. K., A. J. W., C. H., K. M. M., V. K. A., A. R. B., R. C. M., and V. B. O. conceptualization; A. K. and P. D. K. data curation; A. K., P. D. K., M. A., A. J. W., C. H., B. H., J. C., M. S., W. E. B., and R. C. M. formal analysis; A. K., K. M. M., V. K. A., A. R. B., and V. B. O. supervision; A. K. and A. R. B. validation; A. K., P. D. K., M. A., A. J. W., C. H., B. H., J. C., V. J. T., M. S., W. E. B., A. R. B., R. C. M., and V. B. O. investigation; A. K., P. D. K., A. J. W., C. H., B. H., J. C., V. J. T., M. S., W. E. B., A. R. B., R. C. M., and V. B. O. methodology; A. K., A. R. B., R. C. M., and V. B. O. writing-original draft; A. K., P. D. K., M. A., A. J. W., C. H., B. H., J. C., K. M. M., V. J. T., M. S., V. K. A., W. E. B., A. R. B., R. C. M., and V. B. O. writing-review and editing; K. M. M., A. R. B., and V. B. O. project administration; A. R. B. resources; R. C. M. and V. B. O. funding acquisition; R. C. M. visualization.

## Supplementary Material

Supporting Information

## Footnotes

3 The abbreviations used are:

PG: prostaglandin
COX: cyclooxygenase
DXA_3_: 8-hydroxy-9,11-dioxolane eicosatetraenoic acid
8,9–11,12-DiEp-13-HEDE or DiEpHEDE: 8,9–11,12-diepoxy-13-hydroxyeicosadienoic acid
11-HpETE: 11-hydroperoxy-eicosatetraenoic acid
EET: epoxyeicosatrienoic acid
LOX: lipoxygenase
UPLC: ultraperformance LC
AA: arachidonic acid
fMLP: formylmethionylleucylphenylalanine
ACD: acid–citrate–dextrose
IPA: isopropyl alcohol
ESI: electrospray ionization
TEM: temperature
CUR: curtain gas
EP: entrance potential
CXP: collision cell exit potential
DP: declustering potential
CE: collision energy
IHE: interface heater.
