# Supplementary material for: Revising the structure of a new eicosanoid from human platelets to 8,9–11,12-diepoxy-13-hydroxyeicosadienoic acid
Source: J Biol Chem. 2019 May 6;294(23):9225–38. doi: 10.1074/jbc.RA119.008915 (PMC6556573; doi:10.1074/jbc.RA119.008915)
Supplement: Supporting Information [file supp_294_23_9225__index.html]

Revising the structure of a new eicosanoid from human platelets to 8,9-11,12-diepoxy-13-hydroxy-eicosadienoic acid — Generation of a diepoxy-eicosanoid by cyclooxygenase-1 — Revising the structure of a new eicosanoid from human platelets to 8,9–11,12-diepoxy-13-hydroxyeicosadienoic acid — Generation of a diepoxyeicosanoid by cyclooxygenase-1 — Supporting Information 

# Revising the structure of a new eicosanoid from human platelets to 8,9–11,12-diepoxy-13-hydroxyeicosadienoic acid

## Supporting Information

- Supporting Information (to be published online) - Supplementary Schemes and Figure
