# Supplementary material for: Revising the structure of a new eicosanoid from human platelets to 8,9–11,12-diepoxy-13-hydroxyeicosadienoic acid
Source: J Biol Chem. 2019 May 6;294(23):9225–38. doi: 10.1074/jbc.RA119.008915 (PMC6556573; doi:10.1074/jbc.RA119.008915)

Supplemental Figure 1

Endoperoxide product 8*S*-hydroxy-9*S*,11*R*-*cis*-endoperoxy-5*cis*,12*trans*,14*cis*-eicosatrienoate Me ester from 8*R*-LOX reaction with Chiralpak AD chiral column peak 1 (8*S*,9*R*-EET)

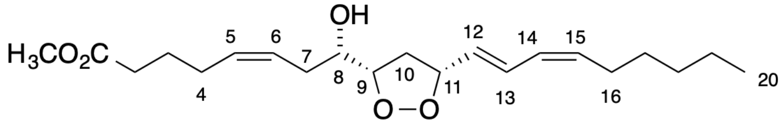

^{1}H-NMR, 600 MHz, C_6D_6, δ 6.60 ppm, 1H, dd, H13; 5.97, 1H, t, H14; 5.53, 3H, m, H12 and H6; 5.40, 1H, dt, H15; 5.47, 1H, dt, H5; 5.35, 1H, dt, H5; 4.50, 1H, q, H11; 3.98, 1H, dt, H9; 3.44, 1H, m, H8; 3.34, 3H, s, CH_3O; 2.25, 2H, m, overlap of H7a, H10a; 2.17, 2H, t, overlap of H7, H11; 2.09, 2H, t, H2; 2.03, 3H, m, overlap of H10b and H16; 1.95, 2H, q, H4; 1.59, 2H, p, H3; 1.15 – 1.35, m, H17, H18, H19; 0.85, 3H, t, H20.

Endoperoxide product 8*R*-hydroxy-9*R*,11*R*-*trans*-endoperoxy-5*cis*,12*trans*,14*cis*-eicosatrienoate Me ester from 8*R*-LOX reaction with Chiralpak AD chiral column peak 2 (8*R*,9*S*-EET)

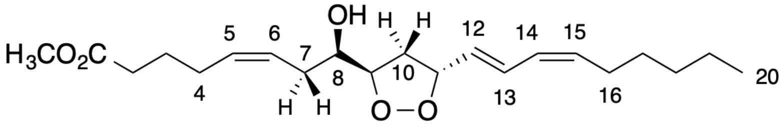

^{1}H-NMR, 600 MHz, C_6D_6, δ 6.59 ppm, 1H, dd, H13; 5.99, 1H, t, H14; 5.55, 1H, dd, H12; 5.49, 1H, dt, H6; 5.42, 1H, dt, H15; 5.35, 1H, dt, H5; 4.59, 1H, dt, H11; 4.02, 1H, dt, H9; 3.35, 4H, m, overlap of CH_3O and H8; 2.36, 1H, ddd, H7a or H10a?; 1.90 – 2.20, m, H2, H7b, H10b, H16, H4; 1.60, 3 or 4H, m, H3 and ?; 1.20 – 1.40, m, H17, H18, H19; 8.85, 3H, t, H20.

Supplemental Scheme 1

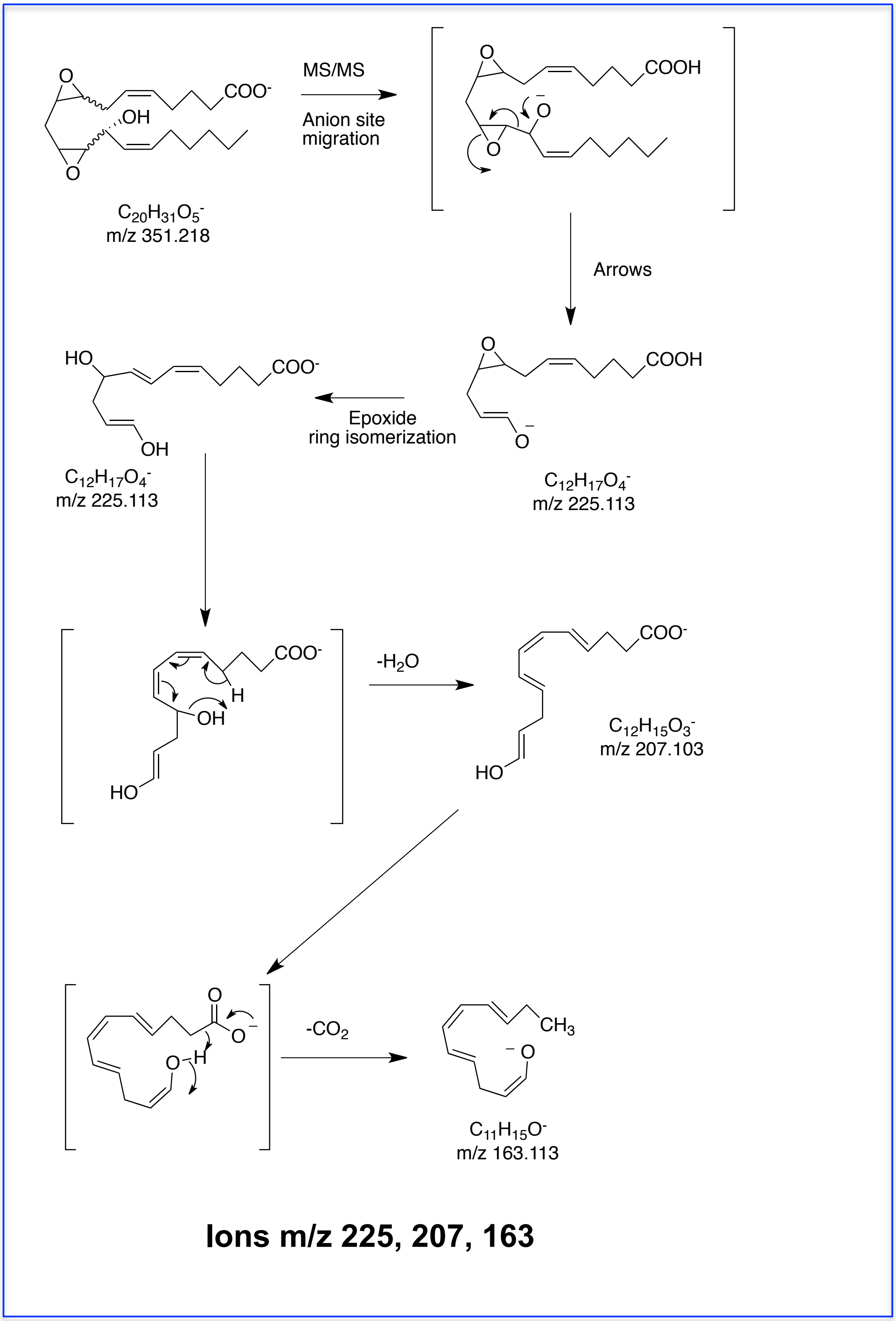

Supplemental Scheme 2

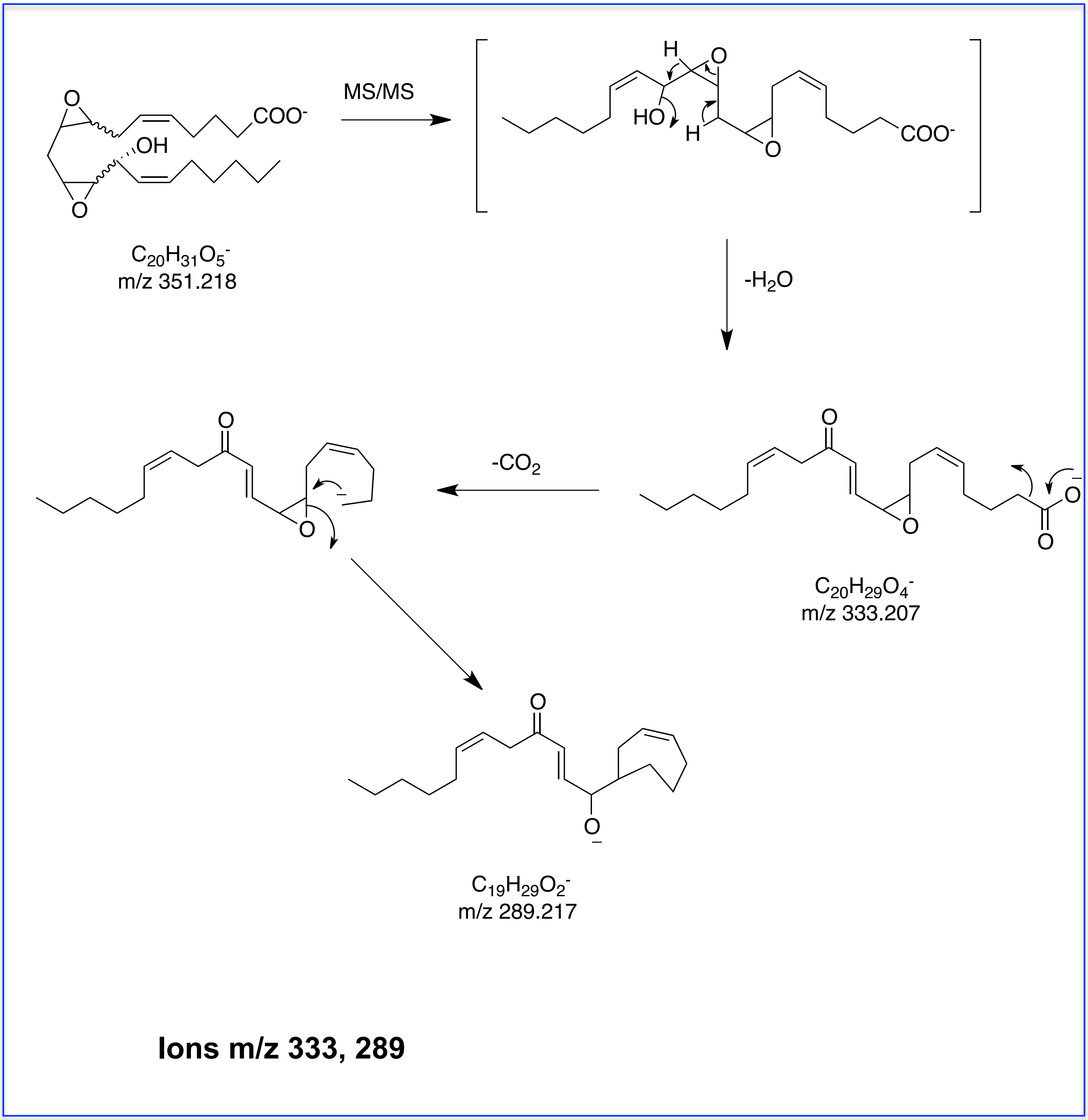

Supplemental Scheme 3

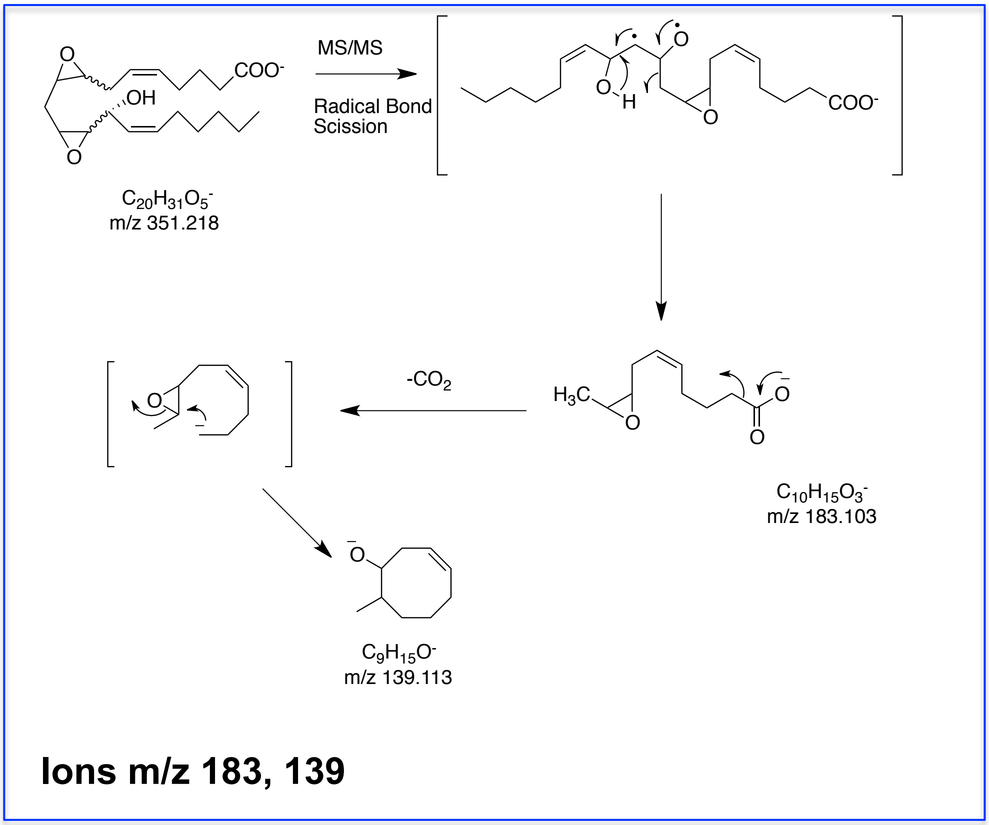

Supplemental Scheme 4

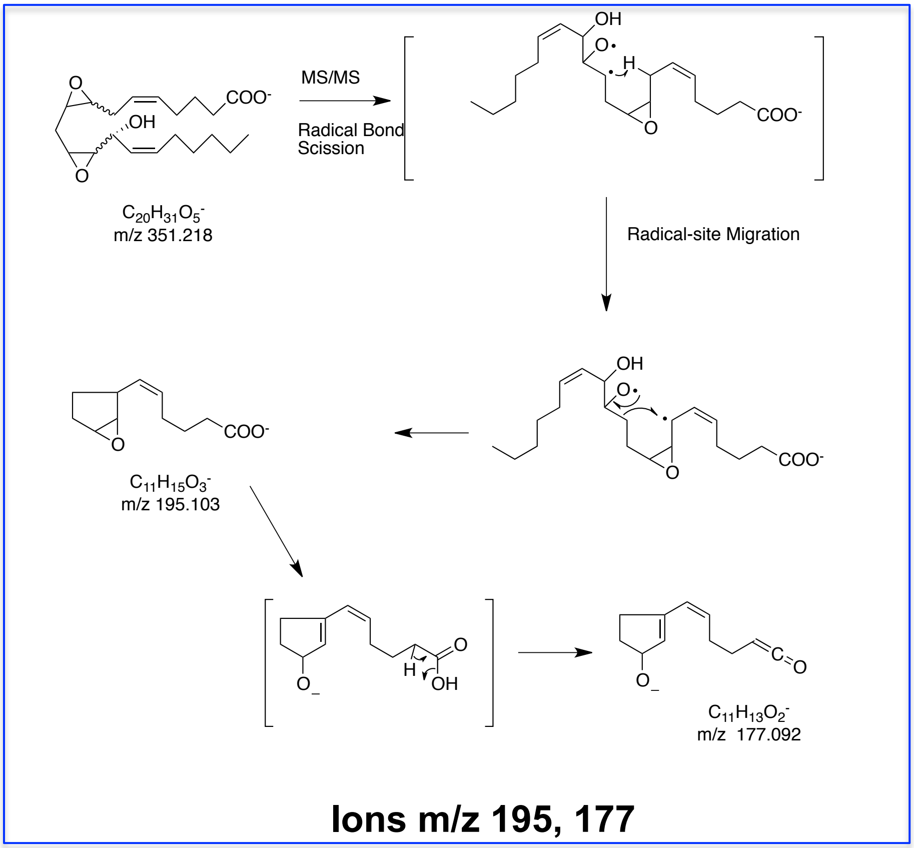

Supplemental Scheme 5

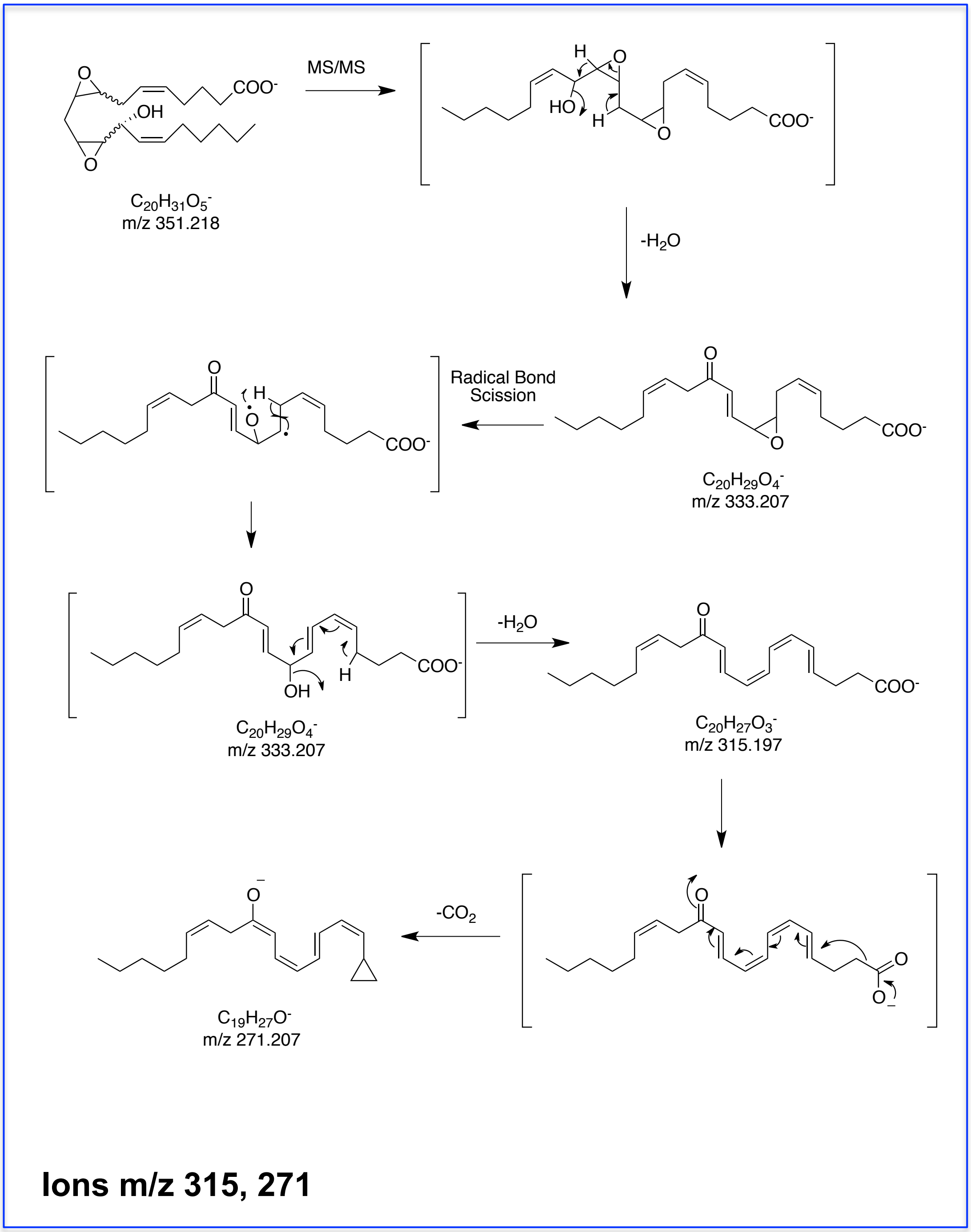

Supplement: Supporting Information [file supp_RA119.008915_144950_2_supp_324597_pqwj4p.pdf]
